# Cross-cultural adaptation of the awareness and beliefs about cancer measure for Hispanics/Latinos living in the United States

**DOI:** 10.3389/fpubh.2024.1351729

**Published:** 2024-09-02

**Authors:** Jennifer Contreras, Chun Wang, Wendy Camelo Castillo, Juan Caicedo, Monica Guerrero Vázquez, Tania Robalino, Aida Hidalgo-Arroyo, Ester Villalonga-Olives

**Affiliations:** ^1^Department of Practice, Sciences, and Health Outcomes Research, University of Maryland School of Pharmacy, Baltimore, MD, United States; ^2^College of Education, University of Washington, Seattle, WA, United States; ^3^Heritage Care, Inc., Riverdale, MD, United States; ^4^Centro SOL, John Hopkins University School of Medicine, Baltimore, MD, United States; ^5^Ezperanza Center, Catholic Charities of Baltimore, Baltimore, MD, United States; ^6^Latino Center for Health, University of Washington, Seattle, WA, United States

**Keywords:** cross-cultural adaptation, cancer knowledge, beliefs, mixed-methods, instrument development, psychometrics, item response theory

## Abstract

**Introduction:**

The purpose of this study is to culturally adapt the Awareness and Beliefs about Cancer (ABC) measure for use in the Hispanic/Latino population living in the United States (US).

**Methods:**

In accordance with Patient Reported Outcomes (PRO) Consortium guidelines for cross-cultural adaptation of measures for content and linguistic validity, we conducted: two forward-translations, reconciliation, two back-translations, revision and harmonization, six cognitive interviews, revision, external expert review, and finalization of the version. We used a mixed methods approach, conducting cognitive interviews with Hispanic/Latino community members while also convening an expert panel of six clinicians, health professionals, and community representatives and including the in the entire process. After cross-culturally adapting the ABC measure, we assessed the psychometric properties of the instrument using item response theory analysis. Item parameters, discrimination and category thresholds, and standard errors were calculated. For each of the adapted subdomains, we used item information curves to report the graphical profile of item effectiveness.

**Results:**

Twenty-two Hispanic/Latino community members were enrolled in cognitive interviews, and Hispanics/Latinos fluent in Spanish completed the measure to assess its psychometric properties. Cognitive interviews revealed opportunities to improve items. Key changes from the original measure include the inclusion of gender inclusive language and an inquiry into e-cigarette use on items related to smoking habits. Psychometric property analyses revealed that the anticipated delay in seeking medical help, general cancer beliefs, and cancer screening beliefs and behaviors subdomains had some slope parameters that were < 1; this implies that those items were not able to adequately discriminate the latent trait and had poor performance.

**Discussion:**

The adapted ABC measure for US Hispanics/Latinos meets content and linguistic validity standards, with construct validity confirmed for cancer symptom recognition and barriers to symptomatic presentation subdomains, but revisions are necessary for others, highlighting the need for ongoing refinement to ensure the cultural appropriateness of instruments.

## 1 Introduction

Cancer is one of the leading causes of death in the United States (US) Hispanic/Latino population (1), and many cancers are diagnosed at advanced stages and have delayed care and treatment (2–4), contributing to lower survival rates (5). Scientific evidence suggests that cancer awareness, knowledge, and beliefs correlate with health-seeking behaviors that impact early detection and treatment outcomes (4–6). To improve treatment and survival outcomes, we need to understand the Hispanic/Latino population's awareness, knowledge, and beliefs about cancer.

Several existing measures assess cancer knowledge and awareness in the general population, although they do not capture information on beliefs toward cancer (7–9). Cancer beliefs, such as *cancer is fatal*, act as barriers to cancer detection and treatment (10–12). Previous studies have shown that Hispanics/Latinos commonly exhibit higher levels of cancer fatalism and lower self-efficacy regarding cancer screening (11, 12). Consequently, this highlights the need for measures to examine cancer awareness, knowledge, and beliefs about cancer in US Hispanics/Latinos. Therefore, measuring cancer awareness, knowledge, and beliefs can provide insight into an individual's motivation to adopt desired health-related behaviors. The Cancer Awareness Measure (CAM) was developed by Cancer Research UK to examine cancer awareness and knowledge (8, 9). Simon et al. modified the CAM to include measurement of beliefs about cancer, resulting in the Awareness and Beliefs about Cancer (ABC) measure, which captures awareness, knowledge, and beliefs about cancer (9). The original ABC measure was created in UK English and has been adapted and used in the US population (13–15). It has been cross-culturally adapted for use in Denmark, Sweden, Norway, and the French-speaking region of Canada. These culturally adapted versions have undergone psychometric testing; however, there is no available Spanish version of the ABC measure.

There is a critical need to understand cancer awareness, knowledge, and beliefs among Hispanics/Latinos with limited English proficiency using measures that are culturally adapted and validated for this population (16). A culturally adapted measure of cancer awareness, knowledge, and beliefs for U.S. Hispanics/Latinos would fill a gap in understanding potential barriers to accessing cancer detection and treatment services. Cross-cultural adaptation is a rigorous process for translating and using culturally relevant content for a target population that includes multiple steps to ensure the adapted measure is understood and maintains its psychometric validity (17–19). An adapted ABC measure would collect information on cancer awareness, knowledge, and beliefs, allowing providers and researchers to identify drivers of health-seeking behaviors in Hispanics/Latinos. Our objective was to culturally adapt the adapted US English-language ABC measure into Spanish for Hispanics/Latinos living in the US.

## 2 Methods

This investigation used a rigorous cross-cultural adaptation of the ABC measure while using sequential exploratory design, which is a mixed-methods approach that is commonly used in instrumentation. The study was conducted from May 2021 to July 2022.

### 2.1 Awareness and beliefs about cancer (ABC) measure

The ABC measure was developed to capture cancer awareness, knowledge, and beliefs and includes a core assessment (32 items), with two optional modules that examine cancer risk factors (13 items) and screening behaviors and beliefs (eight items) (9). The core assessment contains six subdomains: (1) *cancer awareness*, (2) *anticipated delay of seeking medical help*, (3) *cancer symptom recognition*, (4) *self-rated health, access to a doctor and smoking*, (5) *barriers to symptomatic presentation*, and (6) *general cancer beliefs*. The ABC measure was originally designed to be administered over the phone to maximize data collection. Because electronic or internet-based formats are currently widely used, we prepared all items of the adapted ABC measure for implementation via online survey platforms.

### 2.2 Cross-cultural adaptation of the ABC measure

**Figure 1:**
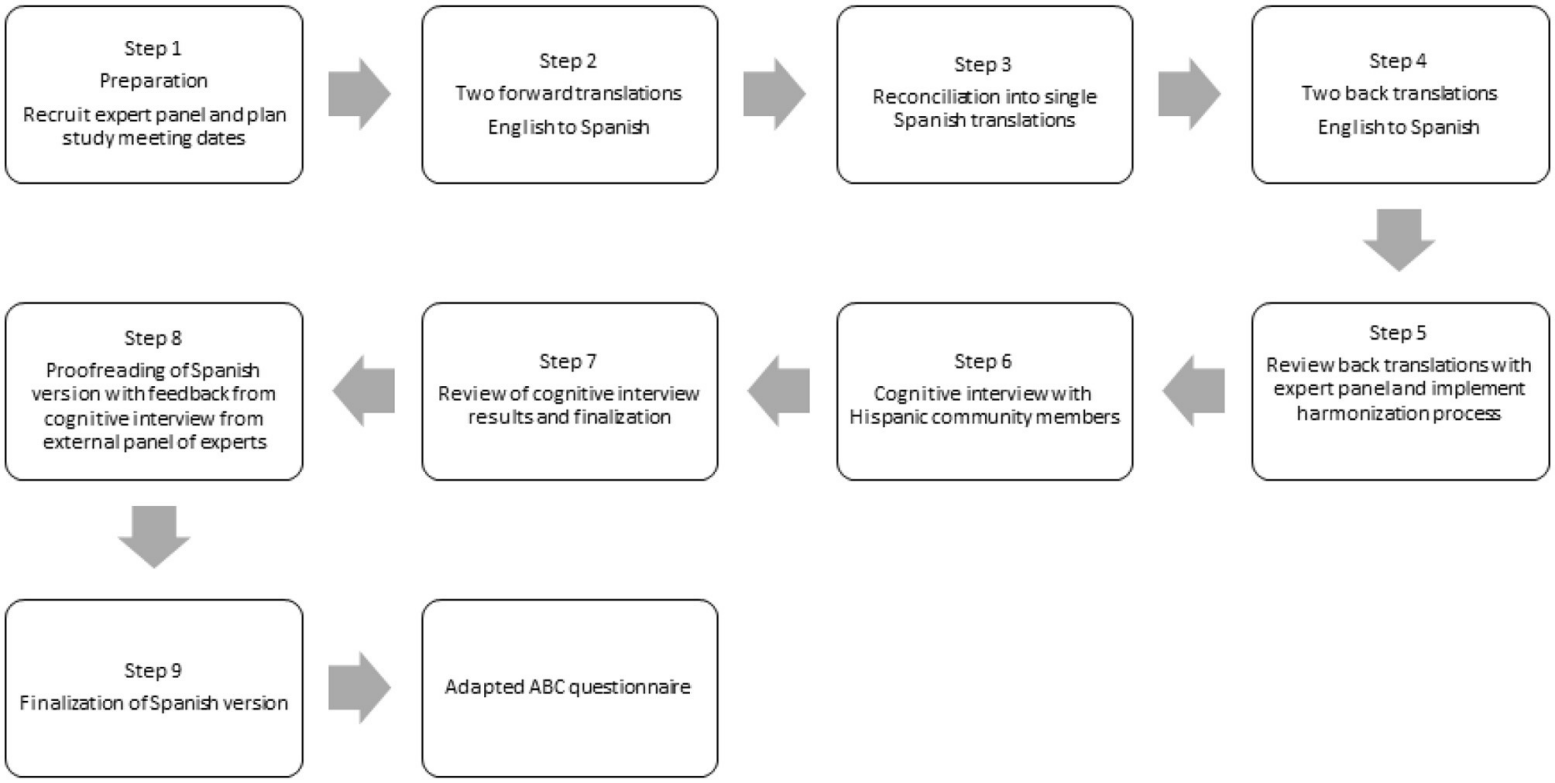
Flow diagram of the translation and cross-cultural adaptation process of the ABC questionnaire.

First, we obtained permission from the original authors of the measure to culturally adapt it into Spanish. Next, we followed the cross-cultural adaptation process of the Patient-Reported Outcome (PRO) Consortium guidelines for achieving content and linguistic validity as follows: two forward-translations, reconciliation, two back-translations, revision and harmonization, cognitive interviews, revision, and external expert review (19). Figure 1 shows the steps of the cross-cultural adaptation process that we followed.

#### 2.2.1 Expert panel

We convened an expert panel to advise about cultural relevance and ensure the content and linguistic validity of the measure. The expert panel (four participants) included three community representatives and health professionals from Maryland (Centro SOL, Esperanza Center, and Heritage Care Inc.) and one community health worker from Washington State. All four experts who self-identified as Hispanic/Latino were originally from Colombia, Mexico, and Ecuador; were fluent in English; had Spanish as their primary language; and have been US residents > 10 years. The purpose of the expert panel was to maintain the conceptual framework of the original measure and prioritize “sense-to-sense” translations over “word-for-word” translations. The panel also assisted with recruitment for cognitive interviews by advertising the study through their respective community organizations.

#### 2.2.2 External expert panel

According to PRO-Consortium guidelines for universal translation, proofreading by native Spanish speakers from various regions or countries is recommended (19). Therefore, we added an external expert panel to ensure that the translations can be understood in different regions of the world that speak Spanish. The inclusion of the external expert panel ensures that the words used in the measure were understood by potential respondents who were born in Latin America and recently migrated to the US. The external expert panel members for the study included health researchers from Argentina, Chile, Costa Rica, Cuba, Puerto Rico, the Dominican Republic, Uruguay, and Venezuela.

#### 2.2.3 Translations and back-translations

Two independent translators who were Spanish native speakers and fluent in English each translated the ABC measure into separate Spanish versions. The study investigators and expert panel then reconciled the two translations into a single Spanish version. Two independent translators then translated the revised Spanish version back into two separate English versions. Finally, the investigators and expert panel reviewed the English versions for inconsistencies and harmonized them to ensure that the correct Spanish translation was used for the adaptation.

#### 2.2.4 Cognitive interviews

To assess the linguistic and content validity of the translated measure, we performed six cognitive interviews with 22 participants from November 2021 to January 2022. The cognitive interviews were performed in two phases: First, we conducted four group cognitive interviews (16 participants) to identify problematic items and improve the adapted measure. Second, we conducted two group cognitive interviews (six participants) to test the revised measure and identify any items that still required adjustment (Appendix 1).

Research staff contacted potential participants via telephone and screened them for eligibility; criteria were being ≥ 18 years old, identifying as Hispanic/Latino, and being fluent in Spanish. An experienced interviewer used a semi-structured discussion guide (Appendix 2), and a trained note-taker was present to record comments during the cognitive interviews. The chief questions asked during the cognitive interviews were, “Please read the item and in your own words explain what the question is asking”, “After reading the question, did you find any words that were difficult to understand?” and “Are you familiar with the word___________?” Both the interviewer and note-taker self-identified as Hispanic/Latino and were fluent in Spanish. The interviews, lasting 90 min each, were conducted with Spanish-speaking participants. Participants received a $30 e-gift card as compensation for their participation and insight. The Institutional Review Board at the University of Maryland, Baltimore approved the study protocol to obtain participants' verbal informed consent during recruitment and again at the beginning of the cognitive interviews.

During the cognitive interviews, to examine which items needed improvement, the interviewer would ask participants to read each item silently, share with the group if it was understood, and explain the question in their own words. In select interviews, the usability of the online platform was assessed. Potential changes to the measure were recorded on an item-tracking matrix by the note-taker. All discussions were audio-recorded and later transcribed verbatim. The transcripts were coded and analyzed using Atlas.ti 22 software while using principles of grounded theory (20). The list of codes from the analysis of the cognitive interviews used *in vivo*, descriptive, and process codes (21, 22). After coding, the codes were organized into central themes that were identified and relevant quotes were selected. Descriptive statistics, encompassing mean, frequencies, and range, were calculated for participant demographic characteristics within the cognitive interview sample using Microsoft Excel.

#### 2.2.5 Summary of cognitive interviews

Table 1 provides the descriptive information for cognitive interview participants. The sample comprised 22 Hispanic/Latino individuals primarily from Latin America with a range of residency durations in the US, extending up to 25 years. The mean age was 43 years (SD = 7), with a predominantly female (81.8%), and a majority of individuals with less than a high school diploma (36.4%) among those who disclosed their education attainment. Analyses of the interviews revealed that most items from the Spanish version of the ABC measure could be understood and answered by the target population. Thematic analysis findings are presented in Appendix 3. Four major themes were identified in the data: (1) *feedback for improvement*, (2) *lack of understanding*, and (3) *cancer knowledge*. Themes and subthemes were consistent across both phases and all cognitive interviews.

**Table 1:** Demographic characteristics of cognitive interview participants (*n* = 22).

Age, range (IQR)	43 (7)
Years in the US, range (IQR)	25 (11.3)
**Sex**, ***n*** **(%)**	
Male	4 (18.2)
Female	18 (81.8)
**Country of origin**, ***n*** **(%)**	
Colombia	1 (4.6)
Ecuador	3 (13.6)
El Salvador	6 (27.3)
Guatemala	2 (9.1)
Honduras	1 (4.6)
Mexico	8 (36.4)
Peru	1 (4.6)
**Education**, ***n*** **(%)**	
Less than high school	8 (36.4)
High school	2 (9.1)
Some College	2 (9.1)
University degree or higher	2 (9.0)
Did not disclose	8 (36.4)

##### 2.2.5.1 Fedback for improvement

The cognitive interviews revealed that the sentence structure of some of the questions could be improved. Participants also suggested replacing a couple of specific words with more common synonyms used in everyday conversations, so the item could be better understood by our target population.

##### 2.2.5.2 Lack of understanding

Participants had a lack of understanding of some of the medical terms used in the *anticipated delay of seeking medical help* subdomain. Consequently, several participants suggested providing a brief explanation for the medical/autonomy terms, using words that are well understood across Latin America. Participants also suggested using alternative Spanish words for one question because they were unsure if the question about breast cancer symptoms was exclusive to women. As a result, alternative words for breast (i.e., “seno/pecho”) that are acceptable to use in clinical settings were used (Appendix 4).

##### 2.2.5.3 Cancer knowledge

The cognitive interviews revealed variability in cancer knowledge within our sample. Several participants were able to explain succinctly how cancer manifests without any presenting symptoms. Other participants had some cancer knowledge but also had some doubts about the accuracy of their knowledge.

#### 2.2.6 Revision of adapted measure by the expert panel

The expert panel reviewed the results of the cognitive interviews and made amendments where necessary. One significant change from the original ABC was to make the language more gender-inclusive, as recommended by the expert panel. Spanish is a gendered language, so wherever the measure used a masculine word (“-o” suffix), the researchers added the corresponding feminine word (e.g., “doctor/doctora”) and both the masculine and feminine articles were included (i.e., “el/ la”). The panel acknowledged that the measure could be more gender inclusive. However, we did not make further changes because guidelines for more gender inclusive language in questionnaire development are still under discussion. Thus, this task remains for future revisions of the questionnaire. Changes based on cognitive interviews included modifying items that asked about smoking habits and rephrasing the items about cancer symptom recognition (Appendix 4). The items that asked about smoking habits were changed to include hookah and e-cigarette use due to the rising popularity of this new modality of smoking, especially among young populations (23).

#### 2.2.7 Revision of adapted measure by external expert panel

To check whether the translations were universally understood, the external expert panel reviewed the preliminary translations and focused on the likelihood the items could be understood by the US Hispanic immigrant population from Latin America. Study investigators reviewed the external panel's results and recommendations and finalized the culturally adapted Spanish version of the ABC measure. The final version, including all questions and their numbers, is available in Appendix 5.

#### 2.2.8 Psychometric properties testing

To examine the psychometric properties of the final version, we had 726 individuals complete the ABC measure in Spanish online. We recruited participants via the Qualtrics research panel, which draws samples from diverse regions across the US and ensures a representative sample of US Hispanics/Latinos. Qualtrics uses detailed profiling attributes to ensure accurate participant selection. We randomly selected participants from those who were eligible; selection criteria included: (a) age 18 years or older; (b) Hispanic/Latino background, either foreign-born or native-born; and (c) fluency in Spanish. Participants received $15 compensation for completing the online instrument. We included both foreign-born and native-born individuals because our measure targeted all Hispanics/Latinos, not just immigrants. Data for the psychometric property testing were collected between February 2022 and May 2022.

We used item response theory (IRT) methods to assess the item-level information for each subdomain of the measure. The IRT is an important method of assessing the psychometric properties of measures because it provides an understanding of how individual items contribute to the overall accuracy of an instrument. The widely used unidimensional IRT models refer to a family of latent variable models that exemplify the underlying relationship between a continuous measured latent trait (i.e., unobservable qualities or characteristics that cannot be observed) and item responses (i.e., the response could be on the nominal, ordinal, or interval/ratio scale). The continuous measured latent trait in this context is cancer awareness, knowledge, and beliefs about cancer. We evaluated all but two of the seven subdomains. The *cancer awareness* subdomain has a single item that is open-ended and requires a written response from a respondent; consequently, it cannot be used for the psychometric property evaluation. Generally, it is not appropriate to use psychometrics to evaluate open-ended questions in a questionnaire due to their qualitative nature, which is not good for traditional quantitative measurement techniques. We did not evaluate *self-rated health access to a doctor and smoking habits* because this subdomain was excluded from the psychometric evaluation of the original measure. Therefore, the following subdomains were evaluated: *anticipated delay in seeking medical help, cancer symptom recognition, barriers to symptomatic presentation, general cancer beliefs, cancer screening beliefs, and behaviors*. Item parameters (e.g., discrimination and category) and standard errors were calculated, and item information curves were created to provide information on the performance of the adapted ABC measure at the item level. An overview of each tool used to test the performance of the adapted ABC measure is summarized in Appendix 6.

Prior to performing the IRT analyses, we reverse-coded the negatively phrased items to check if participants were giving consistent answers. We applied reverse coding to the following subdomains and items: (1) *barriers to symptomatic presentation* subdomain that included items Q24-27b, (2) *general cancer beliefs* for items Q28, Q31-32, and (3) *cancer screening beliefs and behaviors* for items QM3 and QM7. We matched the appropriate IRT model to each item depending on the response type. For the analyses, we used: (1) a graded response model (GRM) for items from the subdomains of *anticipated delay of seeking medical help, barriers to symptomatic presentation, general cancer beliefs*, and *cancer screening beliefs and behaviors*; (2) two-parameter logistic model (2PL) for *cancer symptom recognition*, and (3) nominal response model (NRM) for *anticipated delay of seeking medical help* and *cancer screening beliefs and behaviors*. Appendix 7 shows the purpose of using each model for the subdomains. The “mirt” R package was used to perform the analysis (24).

## 3 Results

### 3.1 Results of the psychometric properties testing

The sample included 726 Hispanics/Latinos who reported Spanish as their primary language and met the study criteria (Table 2). The mean age of respondents was 31.8 years (SD = 11.4 years), most were female (71.1%), and the majority had a household income of < $30,000 (42.7%) and lived in the southern US (42.4%). Half of the sample were foreign-born and approximately one-third had lived in the US for more than 10 years. Educational attainment varied greatly in the sample, from “less than high school” (4.8%) to “high school diploma” (31.8%) to “college degree or greater” (24%).

**Table 2:** Demographic characteristics of ABC respondents (*n* = 726).

Age, mean (SD)	31.8 (11.4)
**Sex**, ***n*** **(%)**	
Female	516 (71.1)
Male	210 (28.9)
**Country of origin**, ***n*** **(%)**	
United States	363 (50)
Outside of the United States	363 (50)
**When did you immigrate to the US?** ***n*** **(%)**	
< 1 year	61 (16.8)
1–2 years ago	33 (9.1)
2–3 years ago	42 (11.6)
3–5 years ago	50 (13.8)
5–10 years ago	69 (19.0)
More than 10 years ago	108 (29.8)
U.S. citizens	363
**Income**, ***n*** **(%)**	
< $30,000	310 (42.7)
$30,000–39,999	130 (17.9)
$40,000–49,999	82 (11.3)
$50,000–59,999	63 (8.7)
$60,000–69,999	37 (5.1)
$70,000–79,999	42 (5.8)
$80,000–89,999	16 (2.2)
$90,000–99,999	20 (2.8)
More than $100,000	26 (3.6)
**Education**, ***n*** **(%)**	
None	7 (1.0)
Less than HS	35 (4.8)
Some HS	97 (13.4)
High school diploma	231 (31.8)
Some college	182 (25.1)
College degree or greater	174 (24.0)
**Primary language**, ***n*** **(%)**	
Spanish	726 (100.0)
**Region**, ***n*** **(%)**	
Midwest	69 (9.5)
Northeast	139 (19.2)
South	307 (42.4)
West	209 (28.9)

Table 3 shows the slope (*a*_*i*_) and threshold (*d*_*x*_) parameters for the subdomains of *anticipated delay in seeking medical help, barriers to symptomatic presentation, general cancer beliefs*, and *cancer screening beliefs and behaviors*. The slope parameters for items Q5-8a in the *anticipated delay in seeking medical help* subdomain were >, which demonstrates that the items performed well and were able to distinguish between individuals with higher or lower levels of delaying seeking medical services (Table 3). However, the standard errors of the threshold parameters *d*_5_ and *d*_6_ for items Q6 and Q7 were >0.3, which is slightly high and suggests that these last two response categories should be combined. Additionally, items Q5.1–Q8a.1 from the anticipated delay of seeking medical help subdomain, which are follow-up questions from items Q5–Q8a, had many response options that were not well endorsed (Appendix 8). Consequently, slope and threshold parameters, as well as standard errors are imprecise for items Q5.1–Q8a.1. The *general cancer beliefs* subdomain with items Q28–33 had several items with slope parameters that were < 1, which indicates that the items differentiate less effectively between individuals with different levels of the latent trait. However, for the *barriers to the symptomatic presentation* subdomain, the slope parameters were >1, which demonstrates that all items in the subdomain performed well (Table 3). Table 4 shows item parameters for the *cancer symptom recognition* subdomain. The results show that all items performed well, indicating that these items were also able to distinguish between individuals with higher or lower levels of knowledge and beliefs about cancer (Table 4).

**Table 3:** Graded response model (GRM) parameters of subdomains with order polytomous categories.

		**Discrimination parameter**	**Threshold 1 (*d_1_*)**	**Threshold 2 (*d_2_*)**	**Threshold 3 (*d_3_*)**	**Threshold 4 (*d_4_*)**	**Threshold 5 (*d_5_*)**	**Threshold 6 (*d_6_*)**
**Anticipated delay in seeking medical help subdomain**								
How long would it take to consult a doctor from the moment you noticed the presence of a persistent cough?	Parameter	1.367	3.557	0.129	−1.057	−2.307	−3.494	−4.382
	SE	0.119	0.202	0.100	0.111	0.146	0.201	0.265
How long would it take to consult a doctor from the moment you noticed the presence of rectal bleeding?	Parameter	1.972	5.602	−1.469	−3.043	−4.234	−5.379	−6.754
	SE	0.188	0.409	0.153	0.220	0.290	0.381	0.551
How long would it take to consult a doctor from the moment you noticed the presence of any breast changes?	Parameter	2.025	5.504	−1.184	−2.834	−4.101	−5.251	−6.989
	SE	0.185	0.389	0.144	0.208	0.276	0.355	0.578
How long would it take to consult a doctor from the moment you noticed the presence of abdominal bleeding?	Parameter	1.534	4.248	−0.025	−1.610	−2.687	−3.688	−4.472
	SE	0.130	0.253	0.105	0.131	0.169	0.217	0.269
How long would it take to consult a doctor from the moment you noticed the presence of a change in the appearance of a mole?	Parameter	1.420	3.649	−0.146	−1.389	−2.567	−3.351	−4.154
	SE	0.124	0.208	0.102	0.120	0.159	0.196	0.247
The *anticipated delay in seeking medical help* subdomain consists of items Q5–Q8, Q8a.								
**Barriers to symptomatic presentation subdomain**								
I would be too embarrassed.	Parameter	2.278	−0.341	−3.585				
	SE	0.229	0.136	0.265				
I would be worried about wasting the doctor's time.	Parameter	2.034	−1.059	−3.224				
	SE	0.210	0.146	0.231				
I would be worried about what the doctor might find.	Parameter	1.706	0.624	−2.090				
	SE	0.155	0.115	0.152				
I am too busy to make time to go to the doctor.	Parameter	1.323	0.303	−2.176				
	SE	0.128	0.099	0.140				
I would be worried about the cost.	Parameter	1.258	0.640	−1.352				
	SE	0.122	0.100	0.113				
I would be worried the doctor would not take my symptoms seriously.	Parameter	1.734	−0.043	−2.411				
	SE	0.161	0.113	0.166				
The *barriers to symptomatic presentation* subdomain consist of items Q24–Q27, Q27a, Q27b, and asked, “could you say if any of these might put you off going to the doctor?” for each of the listed statements.								
**General beliefs about the cancer subdomain**								
These days, many people with cancer can expect to continue with normal activities and responsibilities.	Parameter	0.926	1.708	**-**0.901	**-**2.836			
	SE	0.117	0.117	0.096	0.165			
Most cancer treatment is worse than the cancer itself.	Parameter	**-**0.426	2.481	0.465	**-**1.824			
	SE	0.101	0.140	0.080	0.110			
I would NOT want to know if I have cancer.	Parameter	0.182	0.755	**-**0.582	**-**2.082			
	SE	0.094	0.080	0.078	0.119			
Cancer can often be cured.	Parameter	1.724	1.160	**-**2.069	**-**4.049			
	SE	0.230	0.139	0.189	0.318			
Going to the doctor as quickly as possible after noticing a symptom of cancer could increase the chances of surviving.	Parameter	2.121	**-**0.055	**-**2.892	**-**4.272			
	SE	0.342	0.127	0.313	0.433			
Some people think that a diagnosis of cancer is a death sentence.	Parameter	0.006	1.561	0.027	−1.702			
	SE	0.097	0.098	0.075	0.103			
The *general beliefs about the cancer* subdomain consist of items Q28–Q33, and asked “can you tell me how much you agree or disagree with each item?” for each of the listed statements.								
**Cancer screening beliefs and behaviors**								
I would be so worried about what might be found at breast cancer screening that I would prefer not to have it.	Parameter	2.315	1.433	−1.002	−3.444			
	SE	0.231	0.175	0.161	0.268			
Breast cancer screening is only necessary if I have symptoms.	Parameter	1.999	1.381	−1.105	−3.579			
	SE	0.202	0.160	0.151	0.269			
Breast cancer screening could reduce my chance of dying from breast cancer.	Parameter	0.437	0.648	−1.310	−2.634			
	SE	0.104	0.097	0.110	0.175			
I would be so worried about what might be found at colon cancer screening, that I would prefer not to do it.	Parameter	2.887	1.952	−1.172	−4.441			
	SE	0.314	0.214	0.181	0.381			
Colon cancer screening is only necessary if I have symptoms.	Parameter	1.580	1.739	−0.478	−2.874			
	SE	0.140	0.135	0.108	0.176			
Colon cancer could reduce my chances of dying of colon cancer.	Parameter	0.267	0.768	−1.321	−2.386			
	SE	0.088	0.081	0.092	0.133			
The cancer screening beliefs and behaviors subdomain consists of items Q49–Q51 and Q54–Q55, and asked “can you tell me how much you agree or disagree with each item?” for each of the listed statements.								

**Table 4:** Two-parameter logistic model (2PLM) parameters of cancer symptom recognition subdomain.

**Cancer symptom recognition subdomain**			
		**Slope parameter** ***(a**_1_**)***	**Discriminant parameter** ***(d**_1_**)***
Do you think an unexplained lump or swelling could be a sign of cancer?	Parameter	1.266	−1.383
	SE	0.157	0.124
Do you think unexplained pain could be a sign of cancer?	Parameter	1.567	−1.147
	SE	0.181	0.128
Do you think unexplained bleeding could be a sign of cancer?	Parameter	1.559	−1.591
	SE	0.188	0.145
Do you think persistent cough or hoarseness could be a sign of cancer?	Parameter	2.026	0.009
	SE	0.218	0.123
Do you think a change in bowel or bladder habits could be a sign of cancer?	Parameter	1.445	−1.011
	SE	0.166	0.119
Do you think difficulty in swallowing could be a sign of cancer?	Parameter	1.723	−0.597
	SE	0.189	0.119
Do you think a change in appearance of a mole could be a sign of cancer?	Parameter	1.153	−1.142
	SE	0.144	0.112
Do you think a sore that does not heal could be a sign of cancer?	Parameter	1.502	−0.048
	SE	0.161	0.105
Do you think unexplained night sweats could be a sign of cancer?	Parameter	1.425	0.500
	SE	0.153	0.105
Do you think unexplained weight loss could be a sign of cancer?	Parameter	1.353	−0.901
	SE	0.156	0.112
Do you think unexplained tiredness could be a sign of cancer?	Parameter	1.696	−0.641
	SE	0.187	0.119

The item information curves are provided in Appendices 9–13 for the *anticipated delay of seeking medical help, cancer symptom recognition, barriers to symptomatic presentation, general cancer beliefs*, and *cancer screening beliefs and behaviors* subdomains, respectively. The item information curves graphically show that the subdomains *anticipating delay in seeking medical help, general cancer beliefs*, and *cancer screening beliefs and behaviors* had several items that did not perform well, which is consistent with our observations of large item parameter standard errors as reported in Table 3. The item information curves for the *cancer symptom recognition* (Appendix 10) and *barriers to symptomatic presentation* (Appendix 11) subdomains both show that these items in the scales performed well.

## 4 Discussion

We followed a rigorous process that ensured the linguistic and content validity of our cross-cultural adaptation of the ABC measure in Spanish for US Hispanics/Latinos. The data we gathered from the cognitive interviews demonstrated no significant issues with comprehension of the Spanish adaptation, apart from identifying the need to update the instrument with hookah and e-cigarette use and identifying several technical terms that could be substituted with more colloquial synonyms. The external panel of experts was crucial in providing cultural insight, determining whether to add or keep items identified in the cognitive interviews, and proofreading the adapted measure. According to the IRT analyses, the adapted measure had two subdomains that performed well and were able to distinguish between individuals with higher or lower levels of cancer symptom recognition and acknowledge barriers to symptomatic presentation. The *anticipated delay in seeking medical help, general cancer beliefs*, and *cancer screening beliefs and behaviors* subdomains demonstrated the need for further adjustment to enhance the measure's effectiveness.

Identifying these areas where existing tools may not be optimally suited for the unique needs of Hispanics/Latinos is crucial for refining our approach to measuring cancer awareness, knowledge, and beliefs. By recognizing and addressing these nuances, we contribute to the ongoing effort to improve the cultural relevance of health measures within diverse populations. This nuanced understanding is fundamental for advancing not only the field of cancer research but also for developing more inclusive and effective healthcare interventions tailored to the specific needs of the Hispanic/Latino population.

US Hispanics/Latinos have accounted for ~52% of the total US population growth in the last 10 years (25). Considering the changing demographics of the US population, cross-cultural adaptations of health-related measures into Spanish are in high demand. In recent years, there has been interest in culturally adapting measures for Hispanics/Latinos that measure unmet needs in cancer care (26) and perceptions of human papillomavirus (HPV) (27), a sexually transmitted infection that has been linked to oropharyngeal cancer (OPC). In two separate studies that adapted instruments for Hispanics/Latinos, Cancer Survivor Unmet Needs (CaSUN) (26) and HPV and OPC Knowledge, Perceptions, and Clinical Practices (KPCPs) (27), investigators used a mixed-methods approach similar to ours. However, the CaSUN and the HPV-OPC KPCP investigators provided information for the accuracy of the entire measure and did not provide an analysis at the item level. Compared to the other two measures, our analysis at the item level provides us with more comprehensive information on the performance of the adapted ABC measure. Testing the tool with a more detailed approach likely contributed to our ability to detect problematic items, highlighting the importance of thorough evaluation in cross-cultural adaptation. This increased scrutiny ensures that the future adapted measure not only meets but exceeds the standards for cultural relevance and effectiveness within the Hispanic/Latino community.

Cross-cultural adaptations of measures in Spanish are especially essential for those with limited English proficiency—an estimated one-third of Hispanics/Latinos in the US, according to the Pew Research Center. Among Hispanics/Latinos with limited English proficiency, the vast majority have less than a high school education and/or are foreign-born (28). Evidence suggests that lower educational attainment, low income, and inadequate access to care are associated with cancer fatalism, the belief that death is inevitable after a diagnosis of cancer (10–12). Language barriers, limited cancer knowledge, and fatalistic beliefs about the disease all contribute to the lack of utilization of preventive health services (2, 29, 30).

One potential limitation of our study is the uneven representation of Hispanic/Latino male individuals in our sample for psychometric properties testing. Another limitation is the use of telephone calls to recruit participants for our cognitive interviews. Those who missed the recruitment phone calls were not invited to participate. One additional limitation is that our expert panel did not have representation from all Spanish-speaking countries. Our studies had several strengths, such as the rigorous processes used to adapt the adapted US English-language ABC measure for Hispanics/Latinos living in the US. Our use of an independent expert panel that represented various Latin American countries and had experience in patient involvement was a major strength. As a result, most of the items were understood by all participants in the cognitive interviews. Another strength was that our instrument was designed to be administered online; thus, we modernized as well as adapted the ABC measure. One additional strength is that our psychometric validation sample had similar characteristics in age, educational attainment, and geographic location compared to US Census Bureau data on Hispanics/Latinos (31, 32). Previously, we described our sample as comprising 726 Hispanic/Latino individuals fluent in Spanish. This aligns closely with the 2022 median age of the Hispanic/Latino population reported by the US Census Bureau, which was 30.7 years (32). Additionally, educational attainment in our sample mirrored national trends, with percentages of less than high school (4.8%), some high school (13.4%), high school diploma (31.8%), some college (25.1%), and college degree or greater (24%), closely resembling census data from 2021 (33). Furthermore, our sample distribution across US regions is similar to the geographic trends highlighted by the US Census Bureau, with a significant proportion residing in the US South and West regions, similar to the broader Hispanic/Latino population.

## 5 Conclusion

The Spanish-translated version of the ABC measure for US Hispanics/Latinos meets the standards for content and linguistic validity in culturally adapted measures. Additionally, the psychometric property evaluation demonstrated that the *cancer symptom recognition* and *barriers to symptomatic presentation* subdomains of the adapted ABC measure meet the standards for construct validity. However, there is a need to revise items in the *anticipated delay in seeking medical help, general cancer beliefs*, and *cancer screening beliefs and behaviors* subdomains. The nuanced understanding gained from identifying items that do not perform optimally for Hispanics/Latinos is invaluable for professionals working in the field of cultural adaptations. In future studies, we expect to conduct additional cognitive interviews to improve the construct validity for subdomains with poor performance. We also plan to collapse the response options as necessary, evaluate the performance of the *self-rated health access to a doctor and smoking habits*, and assess the changes made to the adapted measure with a new sample. Importantly, we will also adhere to the soon-to-be-released PRO-Consortium guidelines for the adaptation of measures for online platforms.

### 5.1 Practice implications

Once the ABC measure is modified and tested to ensure its construct validity for the subdomains that need improvement, its use in future studies will provide valuable data for developing interventions designed to increase cancer knowledge in Hispanic/Latino populations in the US. The new ABC measure can also fill a conceptual gap by providing a relatively brief measure to evaluate current programs to increase cancer knowledge in US Hispanics/Latinos. Future studies can potentially evaluate the use of the measure in clinical settings to prioritize subdomains for intervention.

## Data availability statement

The raw data supporting the conclusions of this article will be made available by the authors, without undue reservation.

## Ethics statement

The studies involving humans were approved by University of Maryland Human Research Protections Office. The studies were conducted in accordance with the local legislation and institutional requirements. The Ethics Committee/Institutional Review Board waived the requirement of written informed consent for participation from the participants or the participants' legal guardians/next of kin because the study met the except category for research involving educational tests. The participants provided their verbal informed consent to participate in the study.

## Author contributions

JCo: Data curation, Supervision, Conceptualization, Formal analysis, Project administration, Investigation, Visualization, Writing – original draft, Writing – review & editing. CW: Methodology, Formal analysis, Validation, Investigation, Visualization, Software, Writing – original draft, Writing – review & editing. WC: Investigation, Methodology, Validation, Writing – review & editing. JCa: Methodology, Project administration, Writing – review & editing. MG: Methodology, Project administration, Writing – review & editing. TR: Methodology, Project administration, Writing – review & editing. AH-A: Methodology, Project administration, Writing – review & editing. EV-O: Methodology, Supervision, Conceptualization, Project administration, Investigation, Funding acquisition, Resources, Writing – original draft, Writing – review & editing.
