# Supplementary material for: Cross-cultural adaptation of the awareness and beliefs about cancer measure for Hispanics/Latinos living in the United States
Source: Front Public Health. 2024 Sep 2;12:1351729. doi: 10.3389/fpubh.2024.1351729 (PMC11404523; doi:10.3389/fpubh.2024.1351729)

Supplementary Material

# Supplementary Figures and Tables

***Appendix 1. Summary of the process of the cognitive interview phase (n=22)***

| ***Appendix 2. Cognitive interviews discussion guide*** |
| --- |
| 1. Please read the item and in your own words explain what the question is asking. 2. After reading the question, did you find any words that were difficult to understand? 3. Are you familiar with the word___________?   Probe: in your country of origin, would you use the same word? Would you use a different word?   1. What do you think could be a cause or a risk factor for cancer? 2. Do you think the symptoms listed in this item are a risk factor for cancer? |
| 1. What is your opinion of cancer screenings?   Probe: What effect do you think cancer screenings have on a person’s lifespan? |
| 1. What are the types of cancer treatment that you know of? |
| 1. Imagine you want to find information about cancer for yourself or for someone else. What sources of information do you trust (e.g., Clinician, Newspaper, Cancer League, TV, Government, Industry, Internet)?   Probe: What sources do you find most helpful? |
|  |

| ***Appendix 3. Selected quotes from the themes analyses from cognitive interview transcripts translated in English (n=22)*** | | |
| --- | --- | --- |
| **Themes** | **Subthemes** | **Quotes** |
| Feedback for improvement | Grammar | “I think the part where the other participant describes what persistent means. The first option should be, ‘I would consult the doctor as soon as I would notice’ rather than ‘I would consult the doctor as soon as I notice’” |
|  | Grammar | “I think it’s fine, but perhaps use the word chest or breast instead of mammary gland” |
| Lack of understanding | Medical terminology | “I think everyone would understand breasts. Although, would breasts and chest be the same for a man? The breast is for women, right? And the chest is for men?” |
|  | Medical terminology | “In El Salvador, we would say breast instead of chest” |
|  | Medical terminology | “I don’t think rectal bleeding and bleeding in the rear would be understood the same.” |
|  | Medical terminology | “In the part that says, ‘abdominal bloating’. Does abdominal bloating mean refer to the stomach, tummy, or belly? Would you feel full or stuffed? Tense or swollen?” |
|  | Unclear word | “I understand everything, except for where it says, ‘do you smoke currently either cigarette, including hand-rolled ones?’ Well, I don’t smoke, so I’m not sure what it refers to where it says hand rolled ones” |
|  | Unclear word | “I didn't know they were made by hand. Unless you’re referring to marijuana.” |
| Cancer knowledge | Asymptomatic signs | “‘Cancer manifests itself through many warning signs and symptoms.’ That’s true. I know that cancer can be an asymptomatic disease, which is a silent disease. That many times one does not feel any pain until practically one reaches a final stage, then it is detected.” |
|  | Asymptomatic signs | “Well, I understand that our body is made up of millions and millions of cells. Right? Of which, we do not know in which part of our body the cells can begin to disintegrate, to become damaged and contaminate one another, and thus little by little it is formed. Perhaps it would be the tumor and so on, because later it is detected as a cancer.” |
|  | Lack of cancer knowledge | “Isn’t cancer genetic? Can’t you get it from your family members?” |

| ***Appendix 4. Summary of items from the cross-cultural adaptation of the ABC measures that changed with feedback from the cognitive interviews*** | | | |
| --- | --- | --- | --- |
| **Original version of the ABC item** | **Version of the ABC item prior to cognitive interview sessions** | **Version of the item after the cognitive interview sessions** | **Version of the item after the external panel review** |
| For the list of signs and symptoms, please tell me how long it would take you to go to the doctors from the time you first noticed the symptom. How long it would take you to go to the doctors from the time you first noticed the symptom. | Revise la siguiente lista de signos y síntomas, y piense en cuánto demoraría en consultar al médico en cada caso. ¿Cuánto demoraría en consultar al médico desde el momento en que notó la presencia de los siguientes signos y síntomas? | *No changes made* | *No changes made* |
| Rectal bleeding?  Rectal bleeding means bleeding from the back passage or blood in the bowel motions. | ¿Sangrado rectal?  "Sangrado rectal" significa sangrado que sale del recto (por el trasero/ano) o sangre en las heces (material fecal) | ¿Sangrado rectal?  "Sangrado rectal" significa sangrado que sale del recto (por el ano) o sangre en las heces (excremento) | Al tener sangrado rectal  “Sangrado rectal" significa sangrado que sale del recto (por el ano) o sangre en las heces (excremento) |
| Any breast changes? | ¿Ha notado algún cambio en los senos? | ¿Ha notado algún cambio en los pechos? | Al notar algún cambio en los pechos (endurecimiento, masas, cambios en la piel, secreción, cambio de forma o tamaño, dolor) |
| Abdominal bloating?  Abdominal bloating means your stomach/tummy or belly feels full “stuffed”, tight, or swollen. | ¿Distensión abdominal?  "Distensión abdominal" significa que su estómago/barriga o vientre se siente lleno o repleto, tenso o hinchado. | ¿Barriga hinchada?  "Barriga hinchada" significa que su estómago se siente lleno o replete | Al notar el vientre o estómagos hinchados. |
| Do you think [INSERT WARNING SIGN] could be a sign of cancer?  an unexplained lump or swelling  Yes  No | ¿Cree que [INSERTE EL SIGNO DE ADVERTENCIA] podría ser un signo de cáncer?  Bulto o hinchazón sin motivo aparente  Si  No | ¿Cree que bulto o hinchazón sin motivo aparente podría ser un signo de cáncer?  Si  No | *No changes made* |
| Do you think [INSERT WARNING SIGN] could be a sign of cancer?  A persistent unexplained pain (By persistent, it means that it has lasted for 3-6 weeks)  Yes  No | ¿Cree que [INSERTE EL SIGNO DE ADVERTENCIA] podría ser un signo de cáncer?  Dolor persistente sin motivo aparente (“persistente” significa que ha durado entre 3 y 6 semanas)  Si  No | ¿Cree que dolor persistente sin motivo podría ser un signo de cáncer? ("sin motivo" significa que no se debe a una enfermedad o lesión de la que usted ya tiene conocimiento).  Si  No | *No changes made* |
| Do you think [INSERT WARNING SIGN] could be a sign of cancer?  A persistent difficulty in swallowing (By persistent, it means that it has lasted for 3-6 weeks)  Yes  No | ¿Cree que [INSERTE EL SIGNO DE ADVERTENCIA] podría ser un signo de cáncer?  Dificultad persistente para tragar (“persistente” significa que la tiene desde hace 3 a 6 semanas)  Si  No | ¿Cree que dificultad persistente para tragar/ pasar comida o alimentos podría ser un signo de cáncer?  Si  No | *No changes made* |
| Do you smoke at all these days, either cigarettes, including hand-rolled ones, pipes or cigars?  Yes  No | ¿Fuma actualmente, ya sean cigarrillos, incluidos los hechos a mano, pipas o puros?  Si  No | ¿Fuma actualmente, productos de tabaco, ya sean cigarrillos (incluidos los hechos a mano), cigarrillos electrónicos, los vaporizadores, pipas o puros?  Si  No | ¿Fuma actualmente, productos de tabaco, ya sean cigarrillos (incluidos los hechos a mano), cigarrillos electrónicos, hookah, los vaporizadores, pipas o puros?  Si  No |
| I would be worried the doctor would not take my symptom seriously.  Yes, often  Yes, sometimes  No | Me preocuparía que el/la doctor/a no se tome en serio mi síntoma.  Si, a menudo  Si, a veces  No | Me preocuparía que el medico no tome en serio mi síntoma.  Si, a menudo  Si, a veces  No | *No changes made* |
| Can you tell me how much you agree or disagree with each item?  These days, many people with cancer can expect to continue with normal activities and responsibilities.  Strongly disagree  Tend to disagree  Tend to agree  Strongly agree | ¿Puede decirme en qué medida está de acuerdo o en desacuerdo con cada afirmación?  Actualmente, muchas personas con cáncer pueden esperar seguir con sus actividades y responsabilidades habituales.  Totalmente en desacuerdo  En desacuerdo  De acuerdo  Totalmente de acuerdo | ¿Puede decirme en qué medida está de acuerdo o en desacuerdo con cada afirmación?  Actualmente, muchas personas con cáncer pueden seguir con sus actividades y responsabilidades habituales.  Totalmente en desacuerdo  En desacuerdo  De acuerdo  Totalmente de acuerdo | *No changes made* |
| Can you tell me how much you agree or disagree with each item?  Breast cancer screening could reduce my chance of dying from breast cancer.  Strongly disagree  Tend to disagree  Tend to agree  Strongly agree | ¿Puede decirme en qué medida está de acuerdo o en desacuerdo con cada afirmación?  La prueba de detección de cáncer de mama (seno) podría reducir mis probabilidades de morir de cáncer de mama.  Totalmente en desacuerdo  En desacuerdo  De acuerdo  Totalmente de acuerdo | ¿Puede decirme en qué medida está de acuerdo o en desacuerdo con cada afirmación?  La prueba de detección de cáncer de mama (seno) podría reducir mis probabilidades de morir de cáncer de mama.  Totalmente en desacuerdo  En desacuerdo  De acuerdo  Totalmente de acuerdo | *No changes made* |

| ***Appendix 5. Cross-cultural adaptation of the ABC measure*** | | |
| --- | --- | --- |
| **Question number** | **Subdomain name** | **Original item and adapted version** |
| Q4 | Cancer awareness | *El cáncer se manifiesta a través de muchos síntomas y signos de alerta. Los síntomas son, por ejemplo, malestares o molestias que se experimentan. Los signos de alerta son cambios que se observan en el cuerpo. Por favor, nombre todos los que se le ocurran.* |
| Q5 | Anticipated delay in seeking medical help | *¿Cuánto demoraría en consultar a un/a doctor/a desde el momento en que notara la presencia de los siguientes signos y síntomas? Al tener tos persistente. La tos se considera persistente cuando la tiene desde hace algún tiempo.*  *Consultaría a un/a doctor/a tan pronto lo notara / Hasta 1 semana / Entre 1 y 2 semanas / Entre 2 y 3 semanas / Entre 3 y 4 semanas / Más de un mes / No consultaría a un/a doctor/a* |
| Q5.1 |  | *Si no consultara a un/a doctor/a por la tos persistente, indique el motivo:*   1. *Acudiría a la farmacia en lugar de a un/a doctor/a* 2. *Consultaría a un/a enfermero/a (del consultorio de un/a doctor/a) en lugar de a un/a doctor/a* 3. *Acudiría a un profesional de la salud en una clínica que no requiriera cita previa o a un consultorio de una farmacia. (Por ejemplo, iría a una clínica que no requiriera cita previa como CVS MinuteClinic, a una clínica de atención médica de Walgreens o a una sala de urgencias)* 4. *Acudiría a un profesional de la salud en un hospital en lugar de a un doctor/a* |
| Q6 |  | *¿Cuánto demoraría en consultar a un/a doctor/a desde el momento en que notara la presencia de los siguientes signos y síntomas? Al tener un sangrado rectal. "Sangrado rectal" significa sangrado que sale del recto (por el ano) o sangre en las heces (materia fecal / excrementos).*  *Consultaría a un/a doctor/a tan pronto lo notara / Hasta 1 semana / Entre 1 y 2 semanas / Entre 2 y 3 semanas / Entre 3 y 4 semanas / Más de un mes / No consultaría a un/a doctor/a* |
| Q6.1 |  | *Si no consultara a un doctor/a por tener un sangrado rectal, indique el motivo:*   1. *Acudiría a la farmacia en lugar de a un/a doctor/a* 2. *Consultaría a un/a enfermero/a (del consultorio de un/a doctor/a) en lugar de a un/a doctor/a* 3. *Acudiría a un profesional de la salud en una clínica que no requiriera cita previa o a un consultorio de una farmacia. (Por ejemplo, iría a una clínica que no requiriera cita previa como CVS MinuteClinic, a una clínica de atención médica de Walgreens o a una sala de urgencias)* 4. *Acudiría a un profesional de la salud en un hospital en lugar de a un doctor/a* |
| Q7 |  | *¿Cuánto demoraría en consultar a un/a doctor/a desde el momento en que notara la presencia de los siguientes signos y síntomas? Al notar algún cambio en las mamas o senos (endurecimiento, masas, bultos, cambios en la piel, secreción, cambio de forma o tamaño, dolor).*  *Consultaría a un/a doctor/a tan pronto lo notara / Hasta 1 semana / Entre 1 y 2 semanas / Entre 2 y 3 semanas / Entre 3 y 4 semanas / Más de un mes / No consultaría a un/a doctor/a* |
| Q7.1 |  | *Si no consultara a un/a doctor/a al notar un cambio en las mamas (los senos), indique el motivo:*   1. *Acudiría a la farmacia en lugar de a un/a doctor/a* 2. *Consultaría a un/a enfermero/a (del consultorio de un/a doctor/a) en lugar de a un/a doctor/a* 3. *Acudiría a un profesional de la salud en una clínica que no requiriera cita previa o a un consultorio de una farmacia. (Por ejemplo, iría a una clínica que no requiriera cita previa como CVS MinuteClinic, a una clínica de atención médica de Walgreens o a una sala de urgencias)* 4. *Acudiría a un profesional de la salud en un hospital en lugar de a un doctor/a* |
| Q8 |  | *¿Cuánto demoraría en consultar a un/a doctor/a desde el momento en que notara la presencia de los siguientes signos y síntomas? Al notar el vientre o abdomen hinchados. "Vientre o abdomen hinchados" significa que su vientre o abdomen se siente lleno o repleto.*  *Consultaría a un/a doctor/a tan pronto lo notara / Hasta 1 semana / Entre 1 y 2 semanas / Entre 2 y 3 semanas / Entre 3 y 4 semanas / Más de un mes / No consultaría a un/a doctor/a* |
| Q8.1 |  | *Si no consultara a un/a doctor/a al notar el vientre o abdomen hinchados, indique el motivo:*   1. *Acudiría a la farmacia en lugar de a un/a doctor/a* 2. *Consultaría a un/a enfermero/a (del consultorio de un/a doctor/a) en lugar de a un/a doctor/a* 3. *Acudiría a un profesional de la salud en una clínica que no requiriera cita previa o a un consultorio de una farmacia. (Por ejemplo, iría a una clínica que no requiriera cita previa como CVS MinuteClinic, a una clínica de atención médica de Walgreens o a una sala de urgencias)* 4. *Acudiría a un profesional de la salud en un hospital en lugar de a un doctor/a* |
| Q8a |  | *¿Cuánto demoraría en consultar a un/a doctor/a desde el momento en que notara la presencia de los siguientes signos y síntomas? Al notar un cambio en el aspecto de un lunar. Una zona abultada en la piel que puede aparecer como una pequeña mancha de color marrón o café oscuro.*  *Consultaría a un/a doctor/a tan pronto lo notara / Hasta 1 semana / Entre 1 y 2 semanas / Entre 2 y 3 semanas / Entre 3 y 4 semanas / Más de un mes / No consultaría a un/a doctor/a* |
| Q8a.1 |  | *Si no consultara a un/a doctor/a al notar un cambio en el aspecto de un lunar, indique el motivo:*   1. *Acudiría a la farmacia en lugar de a un/a doctor/a* 2. *Consultaría a un/a enfermero/a (del consultorio de un/a doctor/a) en lugar de a un/a doctor/a* 3. *Acudiría a un profesional de la salud en una clínica que no requiriera cita previa o a un consultorio de una farmacia. (Por ejemplo, iría a una clínica que no requiriera cita previa como CVS MinuteClinic, a una clínica de atención médica de Walgreens o a una sala de urgencias)* 4. *Acudiría a un profesional de la salud en un hospital en lugar de a un doctor/a* |
| Q9 | Cancer symptom recognition | *¿Cree que un bulto una hinchazón sin motivo podría ser un signo de cáncer? ("sin motivo" significa que no se debe a una enfermedad o lesión de la que usted ya tiene conocimiento)*  *Sí / No* |
| Q10 |  | *¿Cree que un dolor persistente sin motivo podría ser un signo de cáncer? ("sin motivo" significa que no se debe a una enfermedad o lesión de la que usted ya tiene conocimiento y “persistente” significa que la tiene desde hace 3 -6 semanas)*  *Sí / No* |
| Q11 |  | *¿Cree que un sangrado sin motivo podría ser un signo de cáncer? ("sin motivo" significa que no se debe a una enfermedad o lesión de la que usted ya tiene conocimiento)*  *Sí / No* |
| Q12 |  | *¿Cree que la tos o la ronquera persistentes podrían ser un signo de cáncer? (“persistente” significa que la tiene desde hace 3 -6 semanas)*  *Sí / No* |
| Q13 |  | *¿Cree que un cambio en los hábitos intestinales o de la vejiga podría ser un signo de cáncer? ("Cambio en los hábitos intestinales y de la vejiga" significa un cambio al defecar o al orinar)*  *Sí / No* |
| Q14 |  | *¿Cree que tener una dificultad persistente para tragar/pasar los alimentos podría ser un signo de cáncer? (“persistente” significa que la tiene desde hace 3 -6 semanas)*  *Sí / No* |
| Q15 |  | *¿Cree que un cambio en la apariencia de un lunar podría ser un signo de cáncer?*  *Sí / No* |
| Q16 |  | *¿Cree que una llaga/herida que no sana podría ser un signo de cáncer?*  *Sí / No* |
| Q17 |  | *¿Cree que tener sudores nocturnos sin motivo aparente podrían ser un signo de cáncer? (“sudores nocturnos” significa sudoración que lo/la despierta y humedece las sábanas y “sin motivo” significa que no se deben a una enfermedad o lesión que ya sabe que tiene)*  *Sí / No* |
| Q18 |  | *¿Cree que la pérdida de peso sin motivo podría ser un signo de cáncer? (“sin motivo” significa que no es intencional ni se debe a una enfermedad o lesión que ya sabe que tiene)*  *Sí / No* |
| Q19 |  | *¿Cree que el cansancio sin motivo podría ser un signo de cáncer? (“sin motivo” significa que no se debe a una enfermedad o lesión que ya sabe que tiene)*  *Sí / No* |
| Q20 | Self-rated health, access to a doctor and smoking | *En general, ¿diría que su salud es...?*  *Muy buena / Buena / Regular / Mala / Muy mala* |
| Q21 |  | *¿Qué tan fácil o tan difícil es para usted ir al/a la doctor/a si tiene un síntoma que cree que podría ser grave?*  *Muy difícil / Bastante difícil / Bastante fácil / Muy fácil* |
| Q22 |  | *¿Fuma actualmente, productos de tabaco, ya sean cigarrillos (incluidos los hechos a mano), cigarrillos electrónicos, hookah, los vaporizadores, pipas o puros?*  *Sí / No* |
| Q23 |  | *¿Alguna vez ha fumado productos de tabaco, ya sean cigarrillos (incluidos los hechos a mano), cigarrillos electrónicos, hookah, los vaporizadores, pipas o puros?*  *Sí / No* |
| Q24 | Barriers to symptomatic presentation | *¿Puede decirnos si alguna de estas razones podría hacerle retrasar la visita a un/a doctor/a? Me daría demasiada vergüenza.**  *Sí, a menudo / Sí, a veces / No* |
| Q25 |  | *¿Puede decirnos si alguna de estas razones podría hacerle retrasar la visita a un/a doctor/a? Me preocuparía hacerle perder el tiempo al/a la doctor/a.**  *Sí, a menudo / Sí, a veces / No* |
| Q26 |  | *¿Puede decirnos si alguna de estas razones podría hacerle retrasar la visita a un/a doctor/a? Me preocuparía lo que pudiera encontrar el/la doctor/a.**  *Sí, a menudo / Sí, a veces / No* |
| Q27 |  | *¿Puede decirnos si alguna de estas razones podría hacerle retrasar la visita a un/a doctor/a? Estoy demasiado ocupado/a para tener tiempo para ir al/a la doctor/a.**  *Sí, a menudo / Sí, a veces / No* |
| Q27a |  | *¿Puede decirnos si alguna de estas razones podría hacerle retrasar la visita a un/a doctor/a? Me preocuparía el costo.**  *Sí, a menudo / Sí, a veces / No* |
| Q27b |  | *¿Puede decirnos si alguna de estas razones podría hacerle retrasar la visita a un/a doctor/a? Me preocuparía que el el/la doctor/a no tome en serio mi síntoma.**  *Sí, a menudo / Sí, a veces / No* |
| Q28 | General cancer beliefs | *Actualmente, muchas personas con cáncer pueden seguir con sus actividades y responsabilidades habituales. ¿Podría indicar en qué medida está de acuerdo o en desacuerdo con esta afirmación?**  *Totalmente en desacuerdo/ En desacuerdo/ De acuerdo/ Totalmente acuerdo* |
| Q29 |  | *El tratamiento para el cáncer es peor que el cáncer en sí mismo. ¿Podría indicar en qué medida está de acuerdo o en desacuerdo con esta afirmación?*  *Totalmente en desacuerdo/ En desacuerdo/ De acuerdo/ Totalmente acuerdo* |
| Q30 |  | *No quisiera saber si tengo cáncer. ¿Podría indicar en qué medida está de acuerdo o en desacuerdo con esta afirmación?*  *Totalmente en desacuerdo/ En desacuerdo/ De acuerdo/ Totalmente acuerdo* |
| Q31 |  | *En muchos casos, el cáncer se puede curar. ¿Podría indicar en qué medida está de acuerdo o en desacuerdo con esta afirmación?**  *Totalmente en desacuerdo/ En desacuerdo/ De acuerdo/ Totalmente acuerdo* |
| Q32 |  | *Consultar al/a la doctor/a lo antes posible al notar un síntoma de cáncer podría aumentar las probabilidades de sobrevivir. ¿Podría indicar en qué medida está de acuerdo o en desacuerdo con esta afirmación?**  *Totalmente en desacuerdo/ En desacuerdo/ De acuerdo/ Totalmente acuerdo* |
| Q33 |  | *Algunas personas piensan que un diagnóstico de cáncer es una sentencia de muerte. ¿En qué medida está de acuerdo o en desacuerdo con que un diagnóstico de cáncer es una sentencia de muerte?*  *Totalmente en desacuerdo/ En desacuerdo/ De acuerdo/ Totalmente acuerdo* |
| Q34 |  | *De cada 10 personas con cáncer de colon, ¿cuántas cree que estarán vivas 5 años después del diagnóstico?*  *Ingrese un número del 1 al 10* |
| Q35 |  | *De cada 10 personas con cáncer de mama (pecho), ¿cuántas cree que estarán vivas 5 años después del diagnóstico?*  *Ingrese un número del 1 al 10* |
| Q36 |  | *De cada 10 personas con cáncer de ovario, ¿cuántas cree que estarán vivas 5 años después del diagnóstico?*  *Ingrese un número del 1 al 10* |
| Q37 |  | *De cada 10 personas con cáncer de pulmón, ¿cuántas cree que estarán vivas 5 años después del diagnóstico?*  *Ingrese un número del 1 al 10* |
| Q38 | Risk | *Ahora piense en la población en general. Durante el próximo año, ¿cuál de estos grupos de personas cree que tiene más probabilidades de recibir un diagnóstico de cáncer?*  *Personas de 30 años/ Personas de 50 años/ Personas de 70 años/ Las personas de cualquier edad tienen la misma probabilidad de recibir un diagnóstico de cáncer* |
| QM1 | Module 1: Cancer screening beliefs and behavior (optional) | *¿Alguna vez se ha hecho una mamografía, que es una prueba de detección del cáncer de mama (seno), en los últimos 5años?*  *Sí / No* |
| QM2 |  | *¿Alguna vez se ha hecho una prueba de detección del cáncer de colon en los últimos 5 años?*  *Sí / No* |
| QM3 |  | *Me preocuparía tanto por el resultado de la prueba de detección del cáncer de mama (seno) que prefiero no hacérmela.**  *Totalmente en desacuerdo/ En desacuerdo/ De acuerdo/ Totalmente acuerdo* |
| QM4 |  | *La prueba de detección de cáncer de mama (seno) solo es necesaria si tengo síntomas.*  *Totalmente en desacuerdo/ En desacuerdo/ De acuerdo/ Totalmente acuerdo* |
| QM5 |  | *La prueba de detección de cáncer de mama (pecho) podría reducir mis probabilidades de morir de cáncer de mama (pecho). ¿Podría indicar en qué medida está de acuerdo o en desacuerdo con esta afirmación?*  *Totalmente en desacuerdo/ En desacuerdo/ De acuerdo/ Totalmente acuerdo* |
| QM6 |  | *Me preocuparía tanto por el resultado de la prueba de detección del cáncer de colon que preferiría no hacérmela.*  *Totalmente en desacuerdo/ En desacuerdo/ De acuerdo/ Totalmente acuerdo* |
| QM7 |  | *La prueba de detección de cáncer de colon podría reducir mis posibilidades de morir de cáncer de colon.**  *Totalmente en desacuerdo/ En desacuerdo/ De acuerdo/ Totalmente acuerdo* |
| QM8 |  | *La prueba de detección del cáncer de colon solo es necesaria si tengo síntomas.*  *Totalmente en desacuerdo/ En desacuerdo/ De acuerdo/ Totalmente acuerdo* |

**The items that were reverse coded prior the psychometric analyses are denoted with an asterisk.*

| ***Appendix 6. Summary of IRT tools used to assess the performance of the ABC measure*** | | |
| --- | --- | --- |
| *IRT Tools* | *Purpose of Tools* | *Interpretation* |
| Discrimination parameter | Discrimination parameters estimate the precision with which each item identifies participants with higher vs. lower levels of the cancer awareness, knowledge, and beliefs. | For binary and ordinal responses (i.e., Likert scale items), positive discrimination means respondents with higher measured latent traits are more likely to endorse the item or choose higher response values, and thresholds indicates how well different response categories are separated. |
| Category thresholds | Category thresholds refer to the points on a scale where people are equally likely to choose one response option over another. | Thresholds that are too close to each other may suggest potential collapsing of adjacent response categories. |
| Item information curves | Item information curves provide a graphical profile of item effectiveness for each of the subdomains of the adapted ABC measure. | Demonstrates how informative the item may be to individuals with limited or adequate cancer knowledge and cancer beliefs. |

| ***Appendix 7. Summary of IRT models used to assess the performance of the ABC measure*** | | |
| --- | --- | --- |
| *IRT Model* | *Purpose of the model* | *Subdomains the model was applied* |
| Graded Response Model (GRM) | GRM is applied for polytomous and ordinal responses such as Likert scale items. This model provides understanding of respondents' abilities or beliefs, especially when dealing with items that have multiple ordered response options. | - Anticipated delay of seeking medical help - Barriers to symptomatic presentation - General cancer beliefs - Cancer screening beliefs and behaviors |
| Two-parameter logistic model (2PL) | 2PL is applied for binary responses. This model provides insight to both item characteristics and respondents' abilities, allowing for a more precise assessment of cancer knowledge. | - Cancer symptom recognition |
| Nominal Response Model (NRM) | NRM is applied for non-ordered response categories. This model helps provide insights into the relationship between cancer awareness, knowledge and beliefs about cancer and the nominal response options for nominal items. | - Anticipated delay of seeking medical help - Cancer screening beliefs and behaviors. |

| ***Appendix 8. NRM Parameters of domain with nominal categories**** | | | | | | | |
| --- | --- | --- | --- | --- | --- | --- | --- |
|  |  | *a_1_* | *ak_1_* | *ak_2_* | *d_1_* | *d_2_* | *d_3_* |
| *If you would not contact your doctor for a persistent cough, please indicate the reason why:* | Parameter | 0.448 | 1.043 | -1.199 | -1.687 | -0.412 | -3.319 |
|  | SE | 0.314 | 1.547 | 1.935 | 1.090 | 0.655 | 1.948 |
| *If you would not contact your doctor for rectal bleeding, please indicate the reason why:* | Parameter | -2.545 | 3.340 | -2.017 | 10.621 | -16.552 | 9.359 |
|  | SE | 4.048 | 3.194 | 4.222 | 17.216 | 22.536 | 15.831 |
| *If you would not contact your doctor for any breast changes, please indicate the reason why:* | Parameter | -0.264 | -14.089 | - | -11.853 | 0.584 | - |
|  | SE | 0.317 | 20.431 | - | 15.450 | 1.285 | - |
| *If you would not contact your doctor for abdominal bleeding, please indicate the reason why:* | Parameter | 0.035 | -0.191 | - | 0.006 | -0.463 | - |
|  | SE | 0.197 | 10.233 | - | 0.537 | 0.625 | - |
| *If you would not contact your doctor for a change in appearance of a mole, please indicate the reason why:* | Parameter | 0.188 | 5.087 | 13.756 | -1.744 | -4.965 | -1.053 |
|  | SE | 0.185 | 5.475 | 13.582 | 1.136 | 2.987 | 0.749 |

**Response categories for domain were: 1- I would go as soon as I would notice; 2- Up to a week; 3- Over 1 up to 2 weeks; 4- Over 3 up to 4 weeks; 5- More than a month; 6- I would go as soon as I noticed; 7- I would not contact my doctor*

***Appendix 9. Item information curve for anticipated delay of seeking medical help subdomain***

**
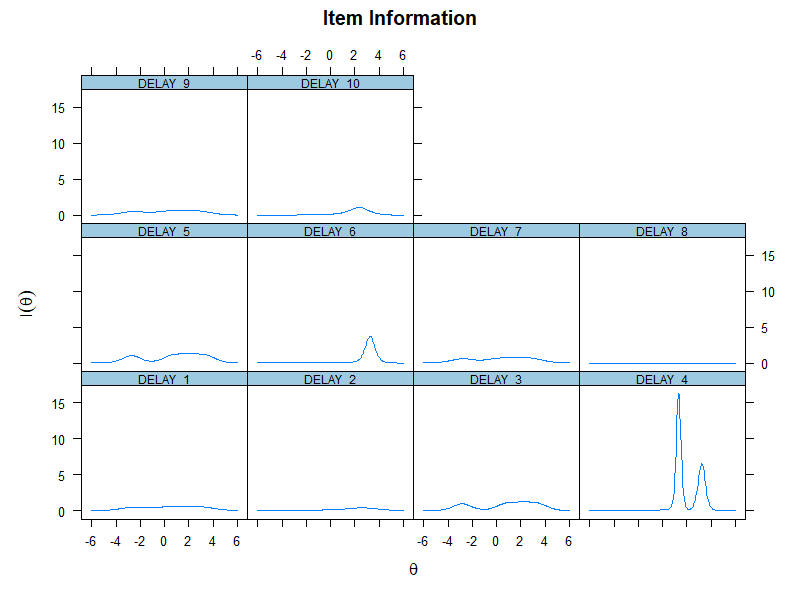
**

***Appendix 10. Item information curve for cancer symptom recognition subdomain***

**
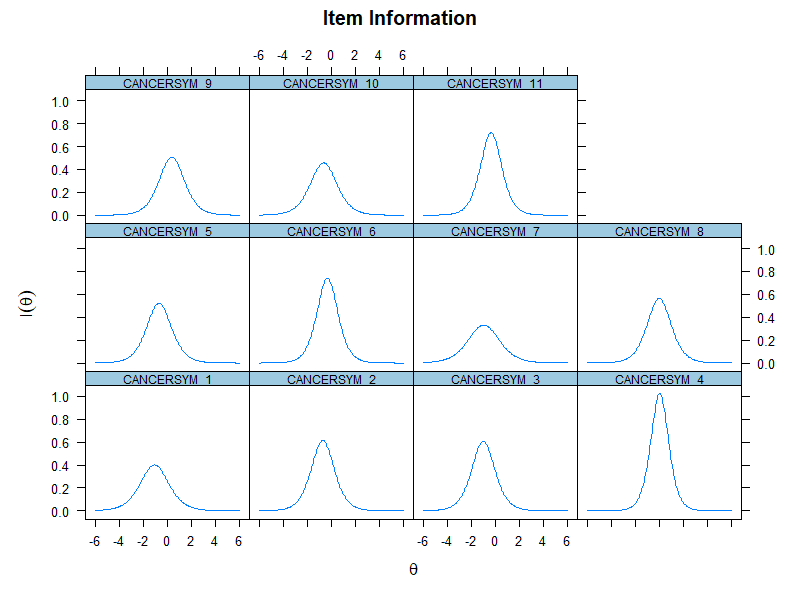
**

***Appendix 11. Item information curve for barriers to symptomatic presentation subdomain***

**
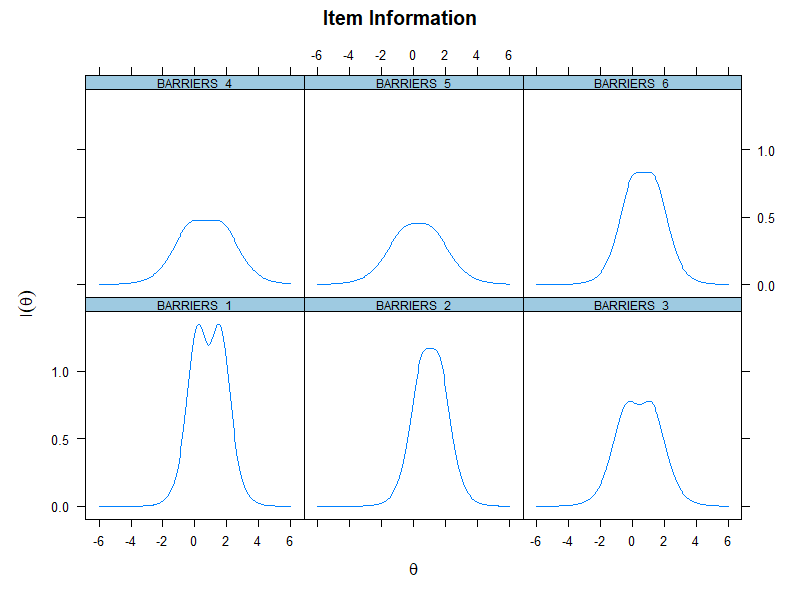
**

***Appendix 12. Item information curve for cancer screening beliefs and behaviors subdomain***

**
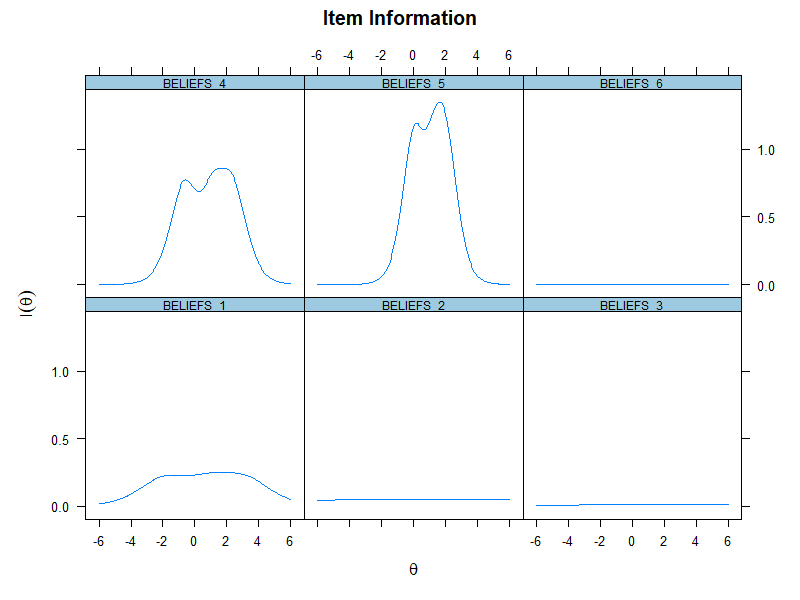
**

***Appendix 13. Item information curve for general cancer beliefs subdomain***


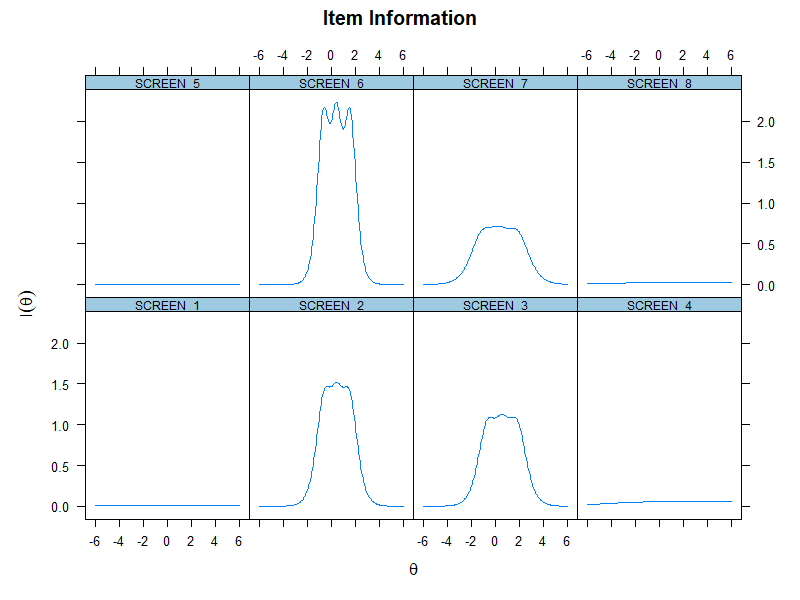

Supplement: Supplementary file 1 [file Data_Sheet_1.docx]
